# Unravelling the Interactions of Magnetic Ionic Liquids by Energy Decomposition Schemes: Towards a Transferable Polarizable Force Field

**DOI:** 10.3390/molecules26185526

**Published:** 2021-09-11

**Authors:** Iván González-Veloso, Nádia M. Figueiredo, M. Natália D. S. Cordeiro

**Affiliations:** LAQV@REQUIMTE, Department of Chemistry and Biochemistry, Faculty of Sciences, University of Porto, Rua do Campo Alegre, 4169-007 Porto, Portugal; up201006255@fc.up.pt

**Keywords:** magnetic ionic liquids, SAPT, energy decomposition, polarisable force field

## Abstract

This work aims at unravelling the interactions in magnetic ionic liquids (MILs) by applying Symmetry-Adapted Perturbation Theory (SAPT) calculations, as well as based on those to set-up a polarisable force field model for these liquids. The targeted MILs comprise two different cations, namely: 1-butyl-3-methylimidazolium ([Bmim]^+^) and 1-ethyl-3-methylimidazolium ([Emim]^+^), along with several metal halides anions such as [FeCl_4_]^−^, [FeBr_4_]^−^, [ZnCl_3_]^−^ and [SnCl_4_]^2−^ To begin with, DFT geometry optimisations of such MILs were performed, which in turn revealed that the metallic anions prefer to stay close to the region of the carbon atom between the nitrogen atoms in the imidazolium fragment. Then, a SAPT study was carried out to find the optimal separation of the monomers and the different contributions for their interaction energy. It was found that the main contribution to the interaction energy is the electrostatic interaction component, followed by the dispersion one in most of the cases. The SAPT results were compared with those obtained by employing the local energy decomposition scheme based on the DLPNO-CCSD(T) method, the latter showing slightly lower values for the interaction energy as well as an increase of the distance between the minima centres of mass. Finally, the calculated SAPT interaction energies were found to correlate well with the melting points experimentally measured for these MILs.

## 1. Introduction

Ionic liquids (ILs) are a class of salts, usually composed of a large organic cation and a small organic or inorganic anion, that have melting points below 100 °C [1]. These compounds have unique properties, such as negligible volatilities, high thermal and electrochemical stabilities, reasonable ionic and viscosity characteristics, wide electrochemical windows and tuneable solvation properties [2]. Moreover, ILs can be fine-tuned by changing their cation or anion structure or by adding other solvents, such as water or alcohols [1,3,4,5].

In 2004, a new subclass of ILs has been discovered [6], so-called magnetic ionic liquids (MILs), which involve the incorporation of transition metal (iron, cobalt, cadmium) or rare earth elements (gadolinium, dysprosium) in the anion or cation structure [7]. The properties of these new MILs are similar to common previous ILs but with the advantage that they strongly respond to external magnetic fields. Indeed, such advantage makes MILs reusable [8,9] and also influences their physicochemical properties, leading for example to lower viscosity and higher self-diffusion coefficients [10,11]. Therefore, the magnetic response allows the possibility of magnetic separation, this being one of the keys that turns them into a better option than other ILs for green chemistry practices, and viscosity manipulation become worthy to be implemented in different industrial processes [12,13].

For example, 1-butyl-3-methylimidazolium tetracloferrate ([Bmim][FeCl_4_]) has been investigated in the removal of various fluorine-containing compounds (benzothiphene (BT), dibenzotipene (DBT) and 4,6-dimethyldibenzothiophene) from a model oil. After being recycled 6 times, the desulfurization activity process achieved an efficiency above 90%, under model conditions [14,15]. Further, 1-octyl-3-methylimidazolium tetrachloroferrate ([Omim][FeCl_4_]) showed high catalytic activity, high selectivity, excellent performance, and recyclability for application in oxidative desulphurisation processes. This MIL was able to de-sulphurise aromatic sulphur compounds, namely DBT, in real diesel fuels with an efficiency of 85%, in 15 min, being reused in at least 7 cycles, without loss of efficacy in the process [16]. Yao et al. show that 1-ethyl-3-methylimidazolium tetrachloroferrate (III) ([Emim][FeCl_4_]) could be used to process the necessary uranium in nuclear power plants [17]. Wang and co-workers found that bis(1-butyl-3-methylimidazolium) stannum tetrachloride salt ([Bmim]_2_[SnCl_4_]) exhibit excellent absorption capacity and selectivity for NH3-containing gases even after five reaction cycles [18]. MILs are also promising compounds in the degradation of polyethylene terephthalate (PET) from waste. For instance, [Bmim] [ZnCl_3_] was reused six times, without losing efficiency in PET conversion, reaching up to 97.9% [19]. In the last decade, several experimental studies exhibit also the potential of applying MILs in DNA extractions [20], CO_2_, NH_3_ and SO_2_ separation [8,21,22], energy harvesting [23] as well as in catalysis applications [24,25]. Rare-earth-based MILs, in addition to their high response to the magnetic field, show high luminescence too, which makes them suitable to use in optical applications [26].

Despite several experimental studies of physicochemical properties of MILs [12], there are still few computational studies at the molecular level, namely by molecular dynamics (MD) simulations to survey their structural and physicochemical properties, or through quantum mechanics calculations to deep insights about their electronic structure and intermolecular forces. Rationalizing the ion-ion interactions and the properties of ILs families is still a challenge and it is an essential asset to target MILs for applications.

Based on quantum mechanics calculations, supermolecular and perturbation approaches allow the analysis of intermolecular interactions at the electronic level between two or more monomers in chemical systems. Within the supermolecular approach, second-order Møller-Plesset perturbation theory (MP2) [27], coupled cluster method (CC) [28,29], and density functional theory (DFT) [30] have been employed to obtain the interaction energy by subtracting the sum of energies of the isolated constituent monomers from the energy of the system. However, over the years, only the CC single-double and perturbative triple [CCSD(T)] level has been considered the “gold standard” of the supermolecular approach for handling these weak interactions [31].

Symmetry-Adapted Perturbation Theory (SAPT) [31], a perturbative approach, has been widely used to unveil non-covalent interaction energies. SAPT directly computes the system interaction energy as a perturbation, starting from the Hamiltonian of its isolated monomers. Aside from the interaction energy, SAPT affords a decomposition of the interaction into physical components of electrostatics, exchange, induction, and dispersion nature. Furthermore, it is possible to improve the SAPT performance by increasing the maximum order of the intra- and intermolecular perturbation used (for example, SAPT2+, SAPT2+3, etc.) or by resorting to a combination of methods, such as CCSD(T), enhancing the treatment of electron correlation [31,32].

Quantifying intermolecular forces plays a crucial role in understanding, predicting, and correlating a series of properties (e.g., solubility, conductivity, or viscosity) of ILs, as well as forward-setting up new polarisable force fields to better capture the dynamics of these liquids. In the last few years, SAPT interaction energies have been primarily computed for IL systems with the purpose to grasp the intermolecular forces that join both fragments together [33,34,35,36]. Recently, however, Padua et al. developed a scheme for computing induction and dispersion terms based on the wavefunction SAPT theory with the aim of reparametrising non-polarisable force fields (FFs). Their strategy eliminates double counting the induction and dispersion contributions included in pairwise effective FFs and adequately upgrade the latter to polarisable models, able of improving both the equilibrium and transport properties of ILs [37]. Izgorodina et al. used also second-order SAPT theory to relate the effect of dispersion components coming from cation-anion interactions with melting points and transport properties for imidazolium and pyrrolidium-based ILs [36]. One should nevertheless notice here that conventional ILs are closed-shell systems, and thus enable an easier application of higher-order SAPT levels of theory.

Efforts have been made to extend the performance of SAPT methods to open-shell systems, for small [38,39,40] and even for big-size systems [32,41,42], but the inclusion of intra-fragment effects is not yet available for open-shell systems, such as MILs, in the commonly employed open-source software package PSI4 [43]. To fill this lack, local energy decomposition (LED) analysis based on the non-perturbative natural orbital local domain (DLPNO)-CCSD(T) method could be applied to large open-shell systems. This method that includes distortion of the molecules, decomposes the energy into two main parts, i.e., the Hartree-Fock (HF) part that includes the intermolecular electrostatic and exchange contributions, and the correlation part that includes the strong and weak pairs contribution as well as the triple correction [44].

Previous experimental studies focussed a lot of effort in investigating ionic liquids but MILs due to their higher difficulty of being synthesized have been more abandoned. However, theoretical studies based on the decomposition of energy are able to provide electronic-level information about the cation-anion interactions in MILs that are unachievable by experimental techniques. As such, they afford a unique ability to interpret MIL properties that are dependent on intermolecular forces in terms of physics-based contributions.

**Figure 1:**
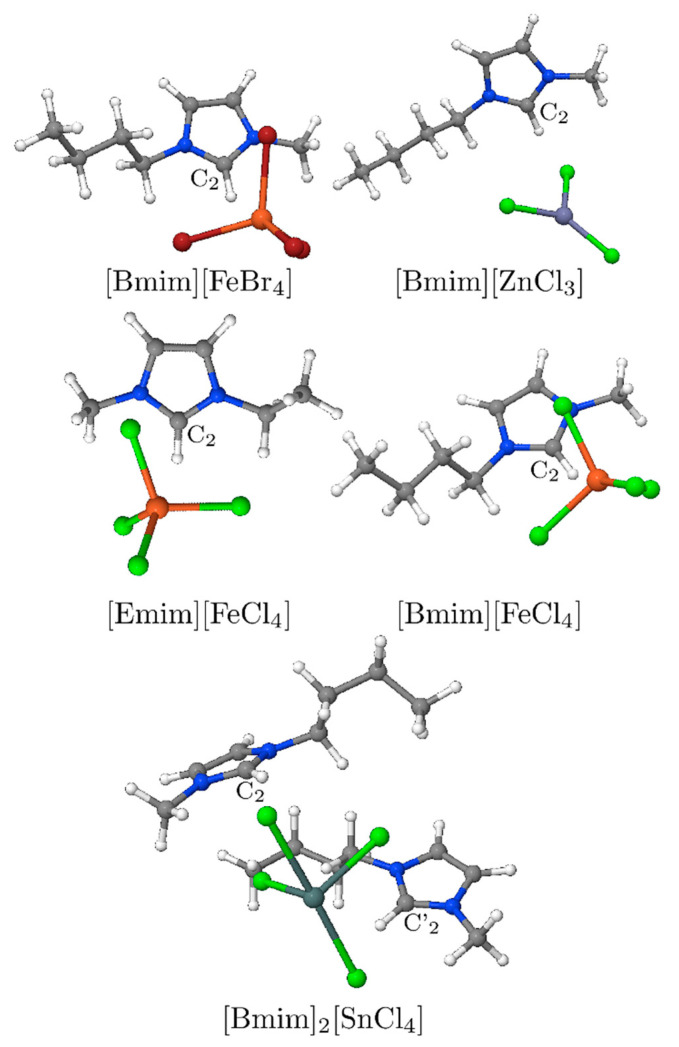
Structures of the different MILs investigated in this work.

This work aims at examining the decomposition of the interaction energy of several metal-containing MILs, i.e., of 1-ethyl-3-methylimidazolium tetrachloroferrate (III) ([Emim][FeCl_4_]), 1-butyl-3-methylimidazolium tetrachloroferrate (III) ([Bmim][FeCl_4_]), 1-butyl-3-methylimidazolium tetrabromoferrate (III) [Bmim][FeBr_4_], 1-butyl-3-methylimidazolium trichlorozincate (II) [Bmim][ZnCl_3_] and bis-(1-butyl-3-methylimidazolium) stannum tetrachloride salt [Bmim]_2_[SnCl_4_] (see Figure 1). These MILs were selected to understand the interaction between their ion pairs as well as to compute the interaction components needed to set-up a new polarisable force field for reliable predicting their properties from molecular simulations. To the best of our knowledge, there is yet no decomposition analysis study of the interaction energy of this type of ionic liquids. [Bmim][FeCl_4_] was the first MIL discovered and it was mainly selected as reference. Meanwhile, [Emim][FeCl_4_] and [Bmim][FeBr_4_] were selected with the purpose to show the effects of changing the number of carbons of the alkyl chain as well as of the halide anionic part. On the other hand, [Bmim][ZnCl_3_] was chosen with the aim to establish weather a planar structure in these MILs produces a significance variance in the interaction energy. Finally, the [Bmim]_2_[SnCl_4_] with a higher difference charge between the anionic and cationic parts was chosen to understand the influence of both the charge and size. Altogether therefore, the chosen MILs will convey us with a detailed interpretation of the cation-anion structure effects at the electronic level by (a) comparing the influence of the metal centre on the cation-anion interactions of the MILs, (b) by inspecting the impact of replacement of the halogen atom in their anion structure; and finally, (c) by analysing the effect of the presence of two cations fragments on their interaction energies.

## 2. Computational Details

Geometries of MIL systems were firstly optimised at the B3LYP-D3(BJ)/aug-cc-PVTZ level following the RIJCOSX [45,46] approximation, and including the dispersion through the Grimme empirical dispersion correction DFT-D3(BJ) [47]. These calculations were performed using the ORCA suite of programs [46]. Following on, a SAPT study was carried out at def2-SVPD level using software PSI4 [43], in which starting from the optimised geometries previously obtained, both fragments of the MIL were separated at a certain distance (0.15 Å) between the centre of mass (CM) keeping fixed their geometries of the latter (see Figure S1, ESI).

As referred to before, the SAPT method is based on perturbation theory and it allows for a decomposition of the interaction energy into different contributions with a physical meaning, i.e., electrostatic (elec), exchange (exch), induction (ind), and dispersion (disp) terms. SAPT is considered to be the state-of-art method for calculating such contributions of the interaction energy, which are e.g., required for the parametrisation of new force polarisable fields [37,48]. At the lowest order in the intermolecular perturbation expansion, the SAPT interaction energy can be written as follows [43]:

(1) $$E_{\mathrm{int}}^{\mathrm{SAPT}}=E_{\mathrm{elec}}^{\left(1\right)}+E_{\mathrm{exch}}^{\left(1\right)}+E_{\mathrm{ind},r}^{\left(2\right)}+E_{\mathrm{exch}-\mathrm{ind},r}^{\left(2\right)}+E_{\mathrm{disp}}^{\left(2\right)}+E_{\mathrm{exch}-\mathrm{disp}}^{\left(2\right)}+\delta_{\mathrm{HF}}^{\left(2\right)}\;,$$

where the superscripts denote the order of expansion of the interaction potential, while the subscript *r* indicates that orbital relaxation effects are included, and the last term is the delta-HF term accounting for higher-order induction effects.

In addition, a different partitioning of the interaction energy was carried out through a local energy decomposition (LED) [44] based on the DLPNO-CCSD(T) method [49,50] implemented in ORCA 4.1. Similar to the previous methodology used in SAPT, both fragments of the MIL are separated at a certain distance between the centre of mass, and the decomposition analysis was conducted at different points to observe the evolution of the interaction energy throughout that distance. The DLPNO-CCSD(T) calculations were performed using the def2-SVPD basis set, and the RIJK approximation with the default basis set employed in ORCA and TightPNO settings. This was done with the purpose to ensure that the most important electron pair in the interactions are included in the CCSD treatment and therefore a correct description of the weak interactions follows. This partition scheme makes it possible to divide the interaction energy into two contributions, i.e., the Hartree-Fock contribution ($\Delta E_{\mathrm{int}}^{\mathrm{HF}}$) and the correlation contribution ($\Delta E_{\mathrm{int}}^{C}$) [51]. Furthermore, the former can be decomposed as:

(2) $$\Delta E_{\mathrm{int}}^{\mathrm{HF}}=\Delta E_{\mathrm{el}-\mathrm{prep}}^{\mathrm{HF}}+E_{\mathrm{elec}}^{\mathrm{HF}}+E_{\mathrm{exch}}^{\mathrm{HF}},$$

where $E_{\mathrm{el}-\mathrm{prep}}^{\mathrm{HF}}$ corresponds to the electronic preparation energy, $E_{\mathrm{elec}}^{\mathrm{HF}}$ to the electrostatic contribution, and $E_{\mathrm{exch}}^{\mathrm{HF}}$ to the exchange contribution. One should notice here that this last energy is always negative because it lowers the repulsion between the same spin electrons, in contrast to what happened in SAPT, for which it is a repulsive component. Regarding the correlation contribution, it can be described as follows:

(3) $$\Delta E_{\mathrm{int}}^{C}=E_{\mathrm{el}-\mathrm{prep}}^{C}+E_{\mathrm{disp}}^{C}+E_{\mathrm{WP}}^{C}+E_{\mathrm{CT}}^{C}+E_{\left(T\right)}^{C},$$

where $E_{\mathrm{el}-\mathrm{prep}}^{C}$ stands for the correlation contribution to the electronic preparation energy, $E_{\mathrm{disp}}^{C}$ for the dispersion energy, $E_{\mathrm{WP}}^{C}$ for the contribution of weak pairs, $E_{\mathrm{CT}}^{C}$ for the charge transfer contribution, and $E_{\left(T\right)}^{C}$ the contribution from triples excitations.

Finally, the non-covalent interaction index (NCI), which is based on a plot of the reduced density gradient at low densities, was employed to visualise the non-covalent interactions [52,53]. Promolecular densities were used because they have shown to yield, at a reduced computational cost, good results in systems containing also high ionic components [54]. As expected, there is a shift towards more negative values when the promolecular densities are used, but both visualisations are quite similar (see Figure S2, ESI). The NCIPLOT4 software suite [53] was applied to perform the NCI analysis of the MILs under study, while the graphic display for the interacting regions between their cation and anion fragments was obtained using the Jmol software [55]. The NCI analysis is essentially based on the heuristic observation that non-covalent interactions in systems pertain to regions of small, reduced density gradient (*s*) at low electronic densities (ρ).

## 3. Results and Discussion

### 3.1. Structure and Stability of Ion Pairs

As conventional ILs, MILs comprise ionic fragments that can consequently assume a diversity of conformations. A careful inspection of the structure of these compounds is extremely important because the ionic pairs can establish several interaction sites in their structure, thus enabling new characteristics and making difficult the comprehension of their dynamics [56,57].

Figure 1 depicts the lowest energy conformations obtained for the [Emim][FeCl_4_], [Bmim][FeCl_4_], [Bmim][FeBr_4_], [Bmim][ZnCl_3_] dimer systems and [Bmim]_2_[SnCl_4_] trimer (see also Figure S1, ESI). As can be seen, a similar behaviour was found for the MILs [Emim][FeCl_4_], [Bmim][FeCl_4_], and [Bmim][FeBr_4_]. Specifically, the position of the anions is in front of the second carbon atom (C2) of the cation—i.e., the carbon atom between the nitrogen atoms in the imidazolium ring, whereas fragments [FeCl_4_] and [FeBr_4_] are found above the plane formed by the imidazolium cation. In the [Bmim][ZnCl_3_] dimer, the anion shows a preference for the C2-H bond, but it appears also closer to the H atoms of the alkyl chain, meaning that [ZnCl_3_] slightly vertically departs from the cation’s plane. This different behaviour could be due to the planar structure of the anion, with thus an increased interaction surface with the cation, while the tetrahedral geometry of the previous anions shows a preference for a higher departure but closer to the 5-membered ring. Following the same trend as the previous MILs, the last system, the [Bmim]_2_[SnCl_4_] trimer adopts a triangular shape in which the Sn atom is found near the C2 atom of the imidazolium ring for both [Bmim] ions (see Figure 1). That is, the [SnCl_4_]^2−^ anion remains above and in front position to the C2-H of the cations. The [Bmim]_2_ cation dimers acquire a rotated position, rotating 90°, considering the C2-H position of the imidazolium ring.

**Figure 2:**
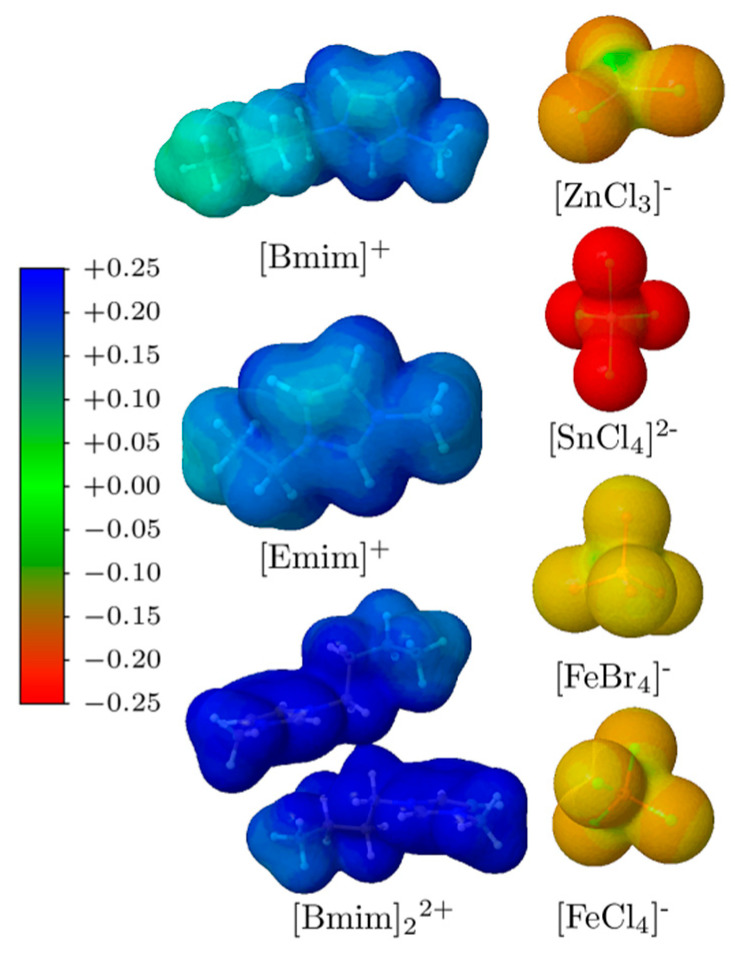
Molecular electrostatic potential (MEP) mapped on an electron density isosurface of 0.004 a.u. for the different fragments in MILs.

The ion-pair interaction sites of a system can be inferred by analysing the charge distribution portrayed by molecular electrostatic potential (MEP) maps. The MEP maps (Figure 2), show the charged regions of the MILs under study. The colour scale applied goes from blue (for the highest positive value of electrostatic potential energy) to red (for the lowest value) for examining the different intensity regions of their potential electrostatic energy. Electropositive regions (richer in protons) have an affinity for attracting electrons in the more electronegative regions and vice versa. As expected, the cations show the most positive electrostatic potential surface sites. In both [Emim] and [Bmim] cations, the positive charge is more localised on the H atoms of the imidazolium ring (blue colour). In the imidazolium ring, the positive charge is delocalised, the C2-H region is more positive (owing to the presence of adjacent N atoms) than the C4-H and C5-H of the imidazolium ring. One can also see that the alkyl chain of the [Bmim] monomer shows a lower electropositive region than its 5-membered ring. The dication shows the most positive molecular electrostatic potential. On the opposite, the negative regions appear localised in the halide atoms (Cl and Br) of the [FeCl_4_] and [FeBr_4_] anions (orange), as well as being more prominent in the [SnCl_4_]^2−^ anion (in red). The conformations adopted by these MILs are ruled out by the C2 region of the imidazolium, because the latter shows an opposite charge region that can establish a good interaction with the negative region of the anionic part (Figure 2).

### 3.2. Non-Covalent Interactions of Ion Pairs from SAPT Level

Hydrogen bonds, π-π stacking, Coulombic, and dispersion forces are the most important interactions reported in ILs [58]. It is also expected that in MIL systems there will be relevant charge transfer effects since they are constituted by open-shell fragments. The decomposition of energy into its components provided by SAPT is therefore essential to investigate these systems.

As shown in Equation (1), the zeroth-order SAPT (SAPT0) theory handles in a simplified manner the monomers, separating the terms of interaction energy into electrostatic, induction, exchange-repulsion, and dispersion components. By applying the same SAPT0 treatment for dispersion, second-order SAPT (SAPT2) appends terms for electrostatics, induction, and exchange up to second-order concerning intramonomer electronic correlation. Intramonomer corrections for dispersion are incorporated at SAPT2+ level and in upper SAPT levels (e.g., SAPT2+3) [31].

**Figure 3:**
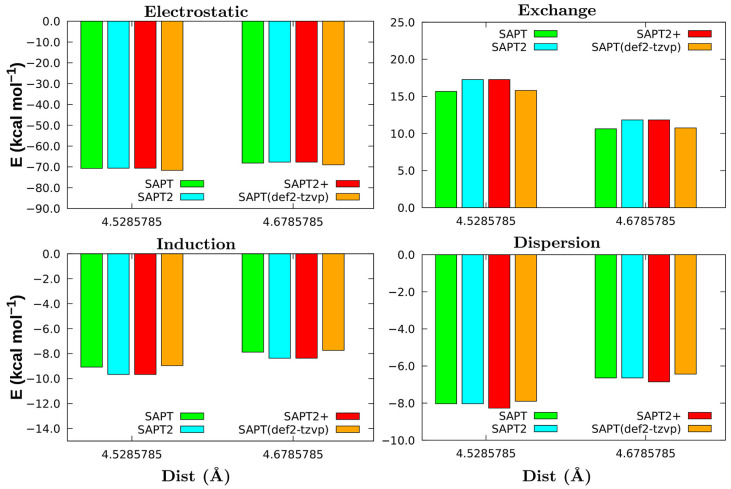
Comparison of different SAPT methodologies for the closed-shell [Bmim][ZnCl_3_] MIL.

A comparison between various SAPT levels of theory was undertaken for the closed-shell [Bmim][ZnCl_3_] MIL, at two different distances around the minimum, to assess the behaviour and the results. Figure 3 shows the electrostatic, exchange, induction, and dispersion terms of the total interaction energy evaluated at both the SAPT0 (green), SAPT0/def2-TZVP (orange), SAPT2 (blue), and SAPT2+ (red) levels. In an overview, one can see that the trend is the same for the two distances represented in Figure 3. For the [Bmim][ZnCl_3_] dimer, the electrostatic component has the main contribution, around –70 kcal mol^−1^. The interaction energy decomposition obtained at SAPT0 and SAPT0 with the def2-TZVP basis set are similar for all contributions, the highest difference being in the electrostatic energy but less than 1 kcal mol^−1^. However, at SAPT2 and SAPT2+ levels, one can notice differences between the energy exchange, induction, and dispersion components. The higher difference occurs in the exchange terms, of about 1 kcal mol^−1^, but for induction, this difference decreases to 0.5 kcal mol^−1^. So, the contribution of these terms increases with the intramolecular correlation introduced at the second-order SAPT level in a more important way than from improving the basis set. As anticipated, the dispersion values obtained with SAPT0 and SAPT2 are equal, as both do not consider intramonomer corrections for the dispersion components. Yet, for SAPT2+, the dispersion term is increased by 0.25 kcal mol^−1^.

To describe the long-range forces for the [Bmim][ZnCl_3_] dimer, the SAPT2+ method certainly exhibits the best performance. Nevertheless, even if energy differences are somewhat visible, SAPT2+ does not produce any change in the trend of interaction contributions for the [Bmim][ZnCl_3_] pair. Further, when the polarised triple-zeta basis set (i.e., def2-TZVP) was applied, that does not improve the system’ interaction energy, thus not justifying its use. Overall, as the results do not produce any change in the trend of [Bmim][ZnCl_3_] interactions, the use of SAPT0 seems sufficient to handle open shell MIL systems. It should be mentioned also here that, higher levels of SAPT for open-shell systems are currently not available in the PSI4 package.

**Figure 4:**
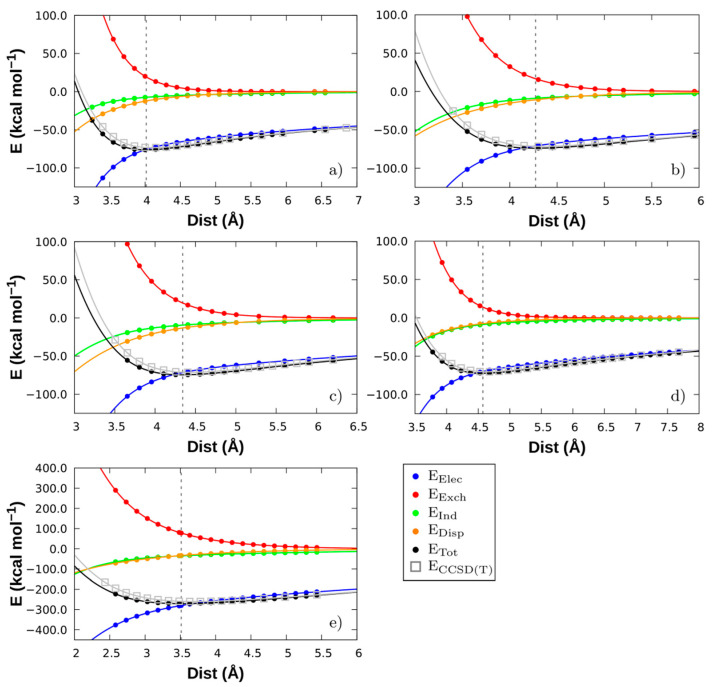
SAPT0 decomposition for an increase distance between the monomers. The LED-DLPNO-CCSD(T) energy is included for comparison purposes. (**a**) [Emim][FeCl_4_], (**b**) [Bmim][FeCl_4_], (**c**) [Bmim][FeBr_4_], (**d**) [Bmim][ZnCl_3_], (**e**) [Bmim]_2_[SnCl_4_].

Hereupon therefore, a SAPT0/def2-SVPD analysis was done for the optimised five fragment-sets corresponding to the following cation-anion pairs: (a) [Emim][FeCl_4_], (b) [Bmim][FeCl_4_], (c) [Bmim][FeBr_4_], (d) [Bmim][ZnCl_3_], and (e) [Bmim]_2_[SnCl_4_]. The decomposition data obtained was then adjusted through a cubic spline extrapolation, the results of which are shown in Figure 4 along with the LED-DLPNO-CCSD(T) energetic terms for comparison purposes.

As seen, in general, the attractive contributions electrostatics (*E*_elec_), induction (*E*_ind_), and London dispersion (*E*_disp_) are negative, whereas the repulsive-exchange (*E*_exch_) forces being the unique component with a positive contribution in the systems, as expected (see Figure 4) [59]. Moreover, the cation-anions combinations under study are dominated by electrostatic attractive components. This tendency was also verified for other conventional ILs [34,60].

Using the geometry found at the minimum of this extrapolation, the SAPT0 interaction energy was obtained, and the results are shown in Table 1. Excluding the [Bmim]_2_[SnCl_4_] that presents a lower centre of mass distance, because the CM for the [Bmim]_2_ is found between both monomers and the [SnCl_4_] can approximate more to this CM (see Figure S1, ESI), the lowest distance between the CM is for the [Emim][FeCl_4_] system—around 4.016 Å. This happens due to the smaller side chain of the [Emim] cation that allows a closer approach to the imidazolium ring. Following on, the [Bmim][FeCl_4_] system has a greater distance (4.273 Å) due to the increase of the carbon chain, from ethyl to a butyl alkyl side chain. In the case of the [Bmim][FeBr_4_], the bigger size of the Br atom is the cause of its higher distance. Finally, the highest distance, 4.574 Å, in the [Bmim][ZnCl_3_] can be explained by the trigonal planar structure of the metallic component. This geometry tries to locate two of the halogen atoms close to the [Bmim]^+^ and for this reason the CM of the [ZnCl_3_] is found farther than in the previous cases. Thus, for dimers with Cl atom in the anion structure, the smaller CM distance is the higher the total interaction energy (*E*_tot_) is, following the decreasing tendency: [Bmim][SnCl_4_] < [Emim][FeCl_4_] < [Bmim][FeCl_4_] < [Bmim][ZnCl_3_].

Concerning the Fe-containing systems, one can observe that the electrostatic energy is ~ −70 kcal mol^−1^. In the [FeCl_4_] ion pair, the main electrostatic component is −75.88 kcal mol^−1^ for the [Emim] cation and decreases about 5 kcal mol^−1^ for the [Bmim] cation. This may be due to the closer contact between the [Emim][FeCl_4_] dimer, arising from a better arrangement of the lower alkyl chain of the cation which reduces the steric effects [59,61]. The greater dispersion effect on the ion pairs with a smaller alkyl chain may be due to the same reason than the higher electrostatic component, i.e., because of the lower CM distance.

Switching halogen in the anion structure, Cl to Br atom, the total interaction energy becomes −73.95 kcal mol^−1^ and −74.60 kcal mol^−1^ for [Bmim][FeCl_4_] and [Bmim][FeBr_4_], respectively. However, the dispersion contribution is larger by ca. 3 kcal mol^−1^ in the [FeBr_4_]-system. This effect can be explained by the increase in the molecular weight of the FeBr_4_ anion, i.e., by replacing Cl with Br atom leads to an increase in the molecule’s size while in turn polarizability increases and the dispersion effect becomes stronger. Both anions assume the same geometry, although, the FeBr_4_ anion is bulkier and electronic density richer compared to FeCl_4_. In [Bmim][FeBr_4_], the intermolecular interactions increase, especially their dispersion and induction components.

**Figure 5:**
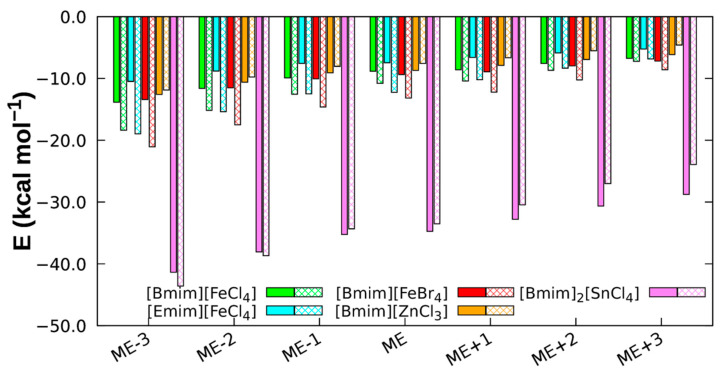
Induction (filled bar) and dispersion (line patterned bar) components obtained through SAPT0 for the MILs. ME is the most stable distance and the signs − or + correspond to a decrease or an increase of the distance between the CM, respectively.

Dispersive and exchange forces have similar magnitude values but opposite signs, and dispersive forces are around 3 kcal mol^−1^ larger than the induction components for Fe-containing dimers. Regarding the van der Waals terms, dispersion forces predominate, at least in the region near the minimum, of Fe-containing MILs (see Figure 5). It is therefore possible to conclude that, the effect of higher dispersion has an impact on the stabilisation of the dimers structures when the anion is in front of C2-H and presents a vertical departure from the imidazolium ring of the cation [62], as it is the case of Fe-based MIL dimers.

In [Bmim][ZnCl_3_], the behaviour is the opposite, i.e., the induction contribution predominates over the dispersion due to the small interaction region between the monomers. Induction and London dispersion show very similar values, −8.69 kcal/mol and −7.58 kcal/mol, respectively.

Two more conformations were considered in the case of the [Bmim][FeCl_4_], i.e., one with the metal anion over the bond C_4_-C_5_ in the imidazolium and the other with it near the alkyl side chain (see Figure S3, ESI). These new conformations were selected to check how a different disposition between the ion pairs affects the stability of the MIL. After performing a similar study than the previous cases, it was found that both conformations follow an analogous trend regarding their components than the optimal conformation (see Figure S4, ESI), though being less stable and displaying higher distances between their centres of mass. The minimum of the interaction energy for the C_4_-C_5_ disposition amounts to a difference of 10 kcal mol^−1^ while the chain disposition to an even higher difference, 35 kcal mol^−1^ (see Table S1). This destabilisation can be explained due to the decrease of all the stabilisation contributions (i.e., electrostatic, induction and dispersion components) and the increase of the exchange at least in the C_4_-C_5_ disposition. This shows that the metal halide anion prefers to stay close to the C_2_ atom due to its more positive region and the better dispersion and induction interactions that are established. Moreover, the chain conformation shows an inversion of the dispersion induction component like in the case of the [Bmim][ZnCl_3_], possible because the anion is far from the imidazolium ring and, in [Bmim][ZnCl_3_], the interaction with this ring was found lower than the ones found for the other MILs. Just like all previous studied cases, the most important contribution for the interaction energy is the electrostatic component, contributing with a similar weight to the interaction energy. In addition, the results reveal that the position of the metal halide anion plays an important role in the stabilisation of these MILs, even if similar energetic trends are observed for other positions rather than the optimal one found—i.e., with the anion over the C_2_ atom (Figure 1).

To conclude, cation-anion interactions between MILs are driven by electrostatic forces. Yet, dispersion forces in MIL systems cannot be ignored; for the same FeCl_4_ anion, dispersion forces increase in the presence of a small alkyl chain and raises with the increasing of the electron density of the anion: *E*_disp_ [FeBr_4_] > *E*_disp_ [FeCl_4_].

Lastly, the [Bmim]_2_[SnCl_4_] has a different behaviour compared to the previous MILs. For the interaction energy analysis of this MIL trimer, the two cations were considered as one unique fragment, and it shows a threefold interaction energy increase with respect to the dimers. The induction and dispersion energetic contributions are threefold of the other MILs, but that is more or less compensated with the value of the exchange energy that increases in the same way. The main reason for its higher interaction energy is thus the electrostatic component, which is the principal contribution stabilising this type of compounds. Such a high increase of the electrostatic component can be explained by the strong negative charge of the [SnCl_4_] as well as the positive charge in [Bmim]_2_ judging from the MEP (see Figure 2). The dimer of [Bmim] cation is composed of two imidazolium ring monomers, i.e., then the number of molecules that allow for charge transfer in the system increases, raising consequently charge transfer effects. Finally, the most stable MIL is the [Bmim]_2_[SnCl_4_] due to the presence of two [Bmim] cations fragments at a shorter distance, thus increasing the favourable contributions to the interaction energy, and more specifically the electrostatic contribution.

Finally, the SAPT interaction energies, in addition to show the stability of the MILs, they can be used to predict their melting points just as Wei et al. has demonstrated for conventional ionic liquids [63]. Considering this, the obtained SAPT interaction energies for the most stable conformations of these MILs were compared to the available melting points experimentally measured. In so doing, a good correlation between both was found (see Figure S5, ESI), thus suggesting that the SAPT interaction energy of MILs could be used to predict their melting points, a very useful utility for discovering new MILs.

### 3.3. Comparison between LED DLPNO-CCSD(T) and SAPT

Moving on to the different partitioning energy carried out by applying the local energy decomposition (LED) scheme at the domain-based local pair natural orbital (DLPNO)–CCSD(T) level. Recently, this framework has been applied for open-shell systems. In this LED scheme, fragment sets of dimer or trimer systems are defined for decomposing its total interaction energy, and the latter being determined by subtracting the energy of the individual fragments from that of the system [44]. Due to the differences between the LED DLPNO–CCSD(T) and the SAPT0 method, it is really difficult to compare quantitatively energy partition values. Notice that even the electrostatic and exchange terms are obtained in a different way than the perturbative method, save for the total interaction energy of the MIL. In fact, as shown in Equations (2) and (3), the LED DLPNO–CCSD(T) decomposition scheme has many more contributions for the interaction energy than SAPT, that is, apart from the electrostatics, exchange, and dispersion ones, it has in addition contributions from weak-pairs, charge transfer, triple excitations, and electronic preparation (see Figure S6, ESI). Table 2 summarises the values obtained for this scheme at fixed fragments geometry at the minimum distance found by SAPT0.

Both contributions of the electron preparation energy are positive (i.e., from HF and correlation) because they stand for the energy investment to obtain the optimal electronic configuration to form the complex, while the other contributions are negative. Another feature of this LED scheme is that the exchange energy contribution is negative, while in the SAPT0 method it is positive. As already mentioned, this is because with this scheme the exchange contribution tends to stabilise the molecule. However, the intermolecular electrostatic energy shows higher values than SAPT0 (over ~1.5 times) but still follow the same trend. The triples correction is particularly small even at a small distance, and its values are very close to zero. One can also compare the LED total dispersion contribution—i.e., obtained by summing up the correlation dispersion and the contribution from weak pairs (Table 2), with the dispersion coming from SAPT (Table 1) [54]. In so doing, one can observe an underestimation for the LED dispersion contribution, though a similar trend is observed regarding the triple correction values for [Bmim]_2_[SnCl_4_] and [Bmim][ZnCl_3_] also displays a small dispersion contribution. Even if the latter is higher than the SAPT one, it is negligible when one compares it with the LED values for charge transfer or electrostatic contributions. Owing to this, it is possible to conclude that, accordingly to both the LED and SAPT results, the main contribution to the interaction energy is due to the different charges of the monomers. Moreover, in all the MILs, it is possible to observe that the charge transfer between the monomers is higher than all the other stabilisation contributions, except the electrostatic component. High charge transfer results are related to the nature of the anion containing electronegative atoms. Furthermore, the fact that the anions are positioned above the cation (see Figure 1) could favour π-interactions with the imidazolium ring's cations. Regarding the charge of the anion, one can see that it shows an increase of its negative value according to the Mülliken population analysis (see Table S2, ESI), opposite to the general behaviour. That can be attributed to the basis set dependence of this scheme, which can indeed produce this type of results [4]. Still, the Natural Population Analysis (NPA) shows a small reduction of the charge in the anion when one cation is present but a higher decrease when there are two of them, showing thus the importance of the charge for the stabilisation of [Bmim]_2_[SnCl_4_]. Finally, it is worth mentioning that the plots in Figure S6 show a higher distance between the CM of the monomers than the SAPT one (around 0.1 Å), as well as a reduction of the interaction energy (see Table S3, ESI).

The two other conformations of the [Bmim][FeCl_4_] show a higher distance between the CM in the minimum and a lower interaction energy than SAPT, i.e., just like the most stable conformation. However, the plot of the LED-DLPNO-CCSD(T) vs. SAPT for the chain conformation shows a flatter tail after the energy minimum when it is compared with other MILs (see Figure S7, ESI), giving a smaller difference for the interaction energy obtained by the two methodologies.

To sum up, both methods show similar equilibrium distances for the MILs, the major difference being the interaction energy in which the SAPT method always leads to higher values (between 2–3 kcal mol^−1^), save for the [Bmim]_2_[SnCl_4_] which shows an increase of 10 kcal mol^−1^ (Table 1). The latter difference can be explained by the tendency of SAPT0 for underestimating the exchange energy. Nevertheless, both methodologies display a similar consistent behaviour for the description of the MILs’ interactions.

### 3.4. NCI Analysis

**Figure 6:**
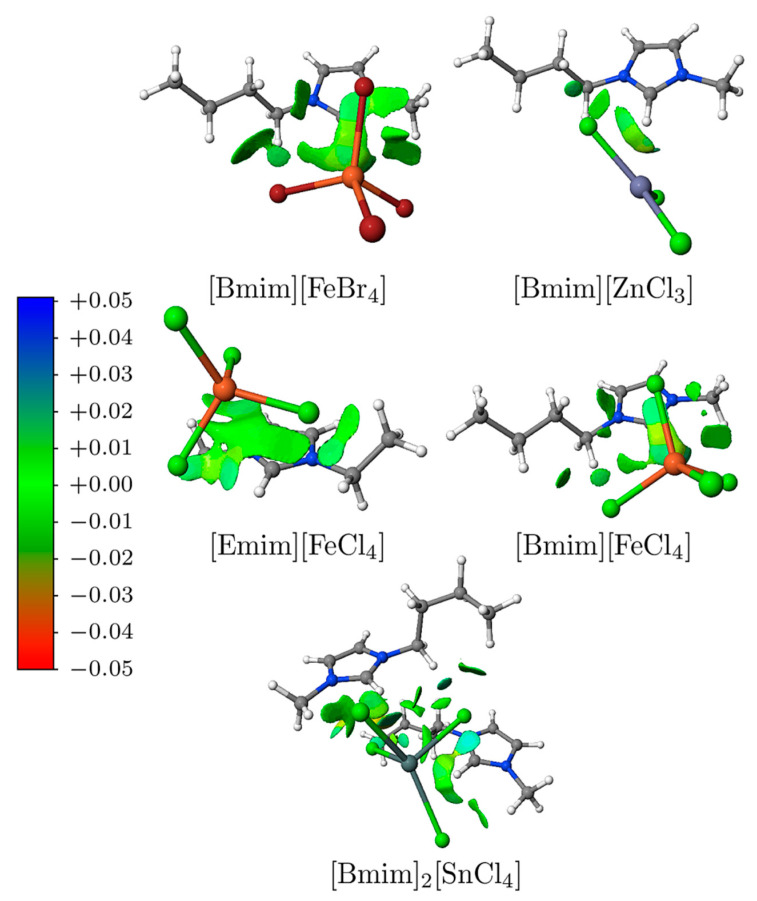
NCIPLOT gradient isosurfaces (0.3 a.u.) for the MILs studied in this work. The colour scales over the range −0.05 to +0.05 a.u.

NCI plots allow for a simple way of visualising the most interesting regions pertaining to non-covalent interactions. Figure 6 shows a three-dimensional representation of the attractive (green colour) and destabilising (blue colour) interaction regions for the MILs. The higher interacting surface is observed for [Bmim]_2_[SnCl_4_] due to the presence of two fragments of [Bmim] cation, in addition to the strongest values of interacting ρ shown in Figure S8 (ESI). As seen in the previous sections, this leads to the highest interaction energies obtained by SAPT and LED DLPNO-CCSD(T). [Emim][FeCl_4_] and [Bmim][FeBr_4_] show a high interacting surface with a predominance of van der Waals (vdW) interactions (green colour) that drives to a high value for the dispersion interaction and, as such, being in good agreement with the SAPT decomposition energy. [Bmim][FeCl_4_] shows a smaller region than the previous MIL but with ρ values away from the vdW region. For this reason, the dispersion contribution unveils a smaller value in accordance with the SAPT0 values, but the final interaction energy is compensated with the presence of stronger interactions, high ρ values. Lastly, the [Bmim][ZnCl_3_] shows the smallest interaction region that leads to the smallest dispersion energy, only in part compensate with higher ρ values. Moreover, this small region helps to explain why in [Bmim][ZnCl_3_] the dispersion contribution has lower importance than the induction one.

Furthermore, the NCI analysis permits obtaining the integrals of the electron density over the active points. If one compares the results of the integrals at *n* = 2.5 (i.e., the same *n* value that Boto et al. found the best correlation for non-covalent interactions [64]) with the interaction energy (see Figure S9, ESI), it is possible to observe that the correlation is inadequate. However, if the integral is obtained for the van der Waals range (i.e., from −0.02 to 0.02 sign(λ^2^)ρ, in which λ stands for the second eigenvalue of the Hessian of the electron density), the correlation fits better with the dispersion energy. Finally, it is worth mentioning that all these results neatly comply with the SAPT values displayed above.

## 4. Conclusions

The geometries of MILs [Emim][FeCl_4_], [Bmim][FeCl_4_], [Bmim][FeBr_4_], [Bmim][ZnCl_3_] and [Bmim]_2_[SnCl_4_] were optimised using DFT methods to obtain their optimal structures. Further, starting from the MIL optimised structures, their interaction energy was examined at different distances between the fragments by employing two methodologies, namely: SAPT0 and LED DLPNO-CCSD(T).

The obtained results indicate that the anion tries to stay close to the C2 atom of the imidazolium ring in all the MILs studied, even in the case of two [Bmim]^+^, the cations try to adopt a geometry that follows this trend. Comparing the interaction energy, it is possible to observe that the MILs with one cation monomer present an interaction energy of about −75 kcal mol^−1^, but the inclusion of two cation monomers produces a higher stabilisation for the MIL, more than the triple of the previous one. The latter is due to the higher electrostatic forces, as a consequence of the charge distribution of their two imidazolium cation monomers. For Fe-containing MILs, the main contribution for the interaction energy is the electrostatic one followed by the attractive dispersion. Nevertheless, in [Bmim][ZnCl_3_] and [Bmim]_2_[SnCl_4_], the induction contributions are higher primarily because the small interaction surface in the vdW region as shown in the NCI analysis.

What is more, comparison with the LED DLPNO-CCSD(T) scheme shows similar values for the interaction energy but always a few kcal mol^−1^ lower than the ones calculated by SAPT0, as well as a slight increase of the distance between the respective centres of mass. Nevertheless, these LED results own a similar trend to that achieved by SAPT, and for this reason, one can conclude that both methodologies can be used indistinctly to study the interaction energy in MILs.

Finally, it is worth mentioning that the present results indicate that the change of the metal atom, the reduction of the aliphatic chain, or the change of the halide atoms produces only slightly changes in the interaction energy of the studied MILs. Nevertheless, an increase of the charge by the presence of two cationic components give rises to an impressive boost in the stability of the MILs. Further studies including a higher number of MILs are however needed to test such a behaviour.

## Figures and Tables

**Table 1:** Contributions to the interaction energy (kcal mol^−1^) using SAPT0. Dist stands for the distance of the minimum interaction energy between the CM. *E*_elec_, *E*_exch_, *E*_ind_, and *E*_disp_ stand for the electrostatic, exchange, induction, and dispersion contributions, respectively. *E*_tot_ stands for the total interaction energy at SAPT0 and *E*_CCSD(T)_ for the total interaction energy at DLPNO-CCSD(T).

System	Dist (Å)	*E*_elec_	*E*_exch_	*E*_ind_	*E*_disp_	*E*_tot_	*E*_CCSD(T)_
[Emim][FeCl_4_]	4.016	−75.88	+19.29	−7.45	−12.24	−76.28	−73.24
[Bmim][FeCl_4_]	4.273	−70.97	+16.63	−8.83	−10.79	−73.95	−71.52
[Bmim][FeBr_4_]	4.342	−71.66	+19.60	−9.36	−13.17	−74.60	−71.09
[Bmim][ZnCl_3_]	4.574	−69.92	+13.94	−8.69	−7.58	−72.25	−70.27
[Bmim]_2_[SnCl_4_]	3.513	−280.07	+77.82	−34.73	−33.52	−270.50	−259.30

**Table 2:** Contributions to the interaction energy (kcal mol^−1^) using DLPNO-CCSD(T). Dist stands for the distance of the minimum interaction energy between the CM. $E_{\mathrm{disp}}^{C}$ stands for the pure dispersion contribution, $E_{\mathrm{WP}}^{C}$ for the contribution from weak pairs, $E_{\mathrm{CT}}^{C}$ for the charge transfer contribution, $E_{\mathrm{elec}}^{\mathrm{HF}}$ for the electrostatic contribution, $E_{\mathrm{exch}}^{\mathrm{HF}}$ for the exchange contribution, $E_{\left(T\right)}^{\mathrm{HF}}$ for the contribution from triple excitations, $E_{\mathrm{el}-\mathrm{prep}}^{\mathrm{HF}}$ for the Hartree-Fock preparation contribution, and $E_{\mathrm{el}-\mathrm{prep}}^{C}$ for the correlation preparation contribution.

**System**	**Dist (Å)**	$E_{\mathrm{disp}}^{C}$	$E_{\mathrm{WP}}^{C}$	$E_{\mathrm{CT}}^{C}$	$E_{\mathrm{elec}}^{\mathrm{HF}}$	$E_{\mathrm{exch}}^{\mathrm{HF}}$	$E_{\left(T\right)}^{\mathrm{HF}}$	$E_{\mathrm{el}-\mathrm{prep}}^{\mathrm{HF}}$	$E_{\mathrm{el}-\mathrm{prep}}^{C}$
[Emim][FeCl_4_]	4.016	−6.20	−0.75	−28.15	−114.87	−10.48	−1.66	+61.62	+27.24
[Bmim][FeCl_4_]	4.273	−5.45	−0.81	−31.21	−111.63	−9.77	−1.51	+58.61	+30.26
[Bmim][FeBr_4_]	4.342	−5.64	−0.97	−31.15	−119.78	−11.63	−1.62	+70.42	+29.27
[Bmim][ZnCl_3_]	4.574	−3.67	−0.65	−31.58	−106.62	−7.75	−1.13	+49.71	+31.42
[Bmim]_2_[SnCl_4_]	3.513	−15.06	−1.73	−102.20	−426.83	−35.73	−4.96	+225.63	+101.57

## Data Availability

The data presented in this study are available on request from the corresponding authors.

## Supplementary Materials

The following are available online, Table S1: Contributions to the interaction energy (kcal mol^−1^ ) using SAPT0.; Table S2: Charge of the MIL anion according to Mulliken and Natural Population Analysis (NPA) at the minimum geometry; Table S3: Comparison between minimum distance and energies between SAPT0 and LED-DLPNO-CCSD(T); Figure S1: Distance between the centre of mass of the different monomers; Figure S2: Comparison between SCF calculation and promolecular calculation in NCIPLOT.; Figure S3: Distance between the centres of mass of different dispositions in [Bmim][FeCl_4_]; Figure S4: The SAPT decomposition for an increase distance between the monomers in [Bmim][FeCl_4_] with different dispositions; Figure S5: Relationship between the SAPT energy and the experimental melting points for different MILs; Figure S6: The LED-DLPNO-CCSD(T) decomposition for an increase distance between the monomers.; Figure S7: TheLED-DLPNO-CCSD(T) decomposition for an increase distance between the monomers in [Bmim][FeCl_4_] with different dispositions; Figure S8: NCI two-dimensional plots for the MILs.; Figure S9: (top) Data fitting between the integral I2.5 and the SAPT interaction energy for the most stable MILs studied. (bottom) Data fitting between the integral I2.5 in the range values for vdW interactions (−0.02 to 0.02 sign(λ_2_)ρ) and the SAPT dispersion energy for the most stable MILs studied.

## Sample Availability

Not available.
