# Supplementary material for: Unravelling the Interactions of Magnetic Ionic Liquids by Energy Decomposition Schemes: Towards a Transferable Polarizable Force Field
Source: Molecules. 2021 Sep 11;26(18):5526. doi: 10.3390/molecules26185526 (PMC8466702; doi:10.3390/molecules26185526)
Supplement: Supplementary file 1 [file molecules-26-05526-s001.zip › molecules-1326248-supplementary.pdf]

Supporting information for

Unravelling the interactions of magnetic ionic liquids by energy decomposition schemes: Towards a transferable polarisable force field

Iván González-Veloso[1*], Nádia M. Figueiredo[1], and M.Natalia D.S. Cordeiro [1*]

[1] *LAQV@REQUIMTE, Department of Chemistry and Biochemistry, Faculty of Sciences, University of Porto, Rua do Campo Alegre, 4169-007 Porto, Portugal E-mail: ivan.veloso@fc.up.pt; ncordeiro@fc.up.pt*

Table S1 Contributions to the interaction energy (kcal mol^{-1}) using SAPT0. Dist is the distance of the minimum interaction energy between the CM, E_{Elec} is the electrostatic contribution, E_{Exch} is the exchange contribution, E_{Ind} is the induction contribution, E_{Disp} is the dispersion contribution, E_{Tot} is the total interaction energy in SAPT0 and $E_{CCSD(T)}$ is the interaction energy with DLPNO-CCSD(T) 1

Table S2 Charge of the MIL anion according to Mulliken and Natural Population Analysis (NPA) at the minimum geometry. . . 1

Table S3 Comparison between minimum distance and energies between SAPT0 and LED-DLPNO-CCSD(T). 1

Figure S1 Distance between the centre of mass of the different monomers. 2

Figure S2 Comparison between SCF calculation and promolecular calculation in NCIPLOT. 3

Figure S3 Distance between the centre of mass of the different additional configurations of [Bmim][$FeCl_4$] 4

Figure S4 SAPT decomposition for an increase distance between the monomers in [Bmim][$FeCl_4$] with different dispositions. 5

Figure S5 Relationship between SAPT energy and the experimental melting points for different MILs. Relationship between SAPT energy and the experimental melting points for different MILs. 5

Figure S6 The LED-DLPNO-CCSD(T) decomposition for an increase distance between the monomers. The SAPT0 energy is included with the purpose to compare the results. a) [Emim][$FeCl_4$], b) [Bmim][$FeCl_4$], c) [Bmim][$FeBr_4$], d) [Bmim][$ZnCl_3$], e) $[Bmim]_2[SnCl_4]$ 6

Figure S7 The LED-DLPNO-CCSD(T) decomposition for an increase distance between the monomers in [Bmim][$FeCl_4$] with different dispositions. 7

Figure S8 NCI two-dimensional plots for the MILs. The dark scale is obtained for the [Bmim][$FeCl_4$] and the other MIL is displaced 0.1 a.u. in s for clarity. 8

Figure S9 (top) Data fitting between the integral I2.5 and the SAPT interaction energy for the most stable MILs studied. (bottom) Data fitting between the integral I2.5 in the range values for VdW interactions (-0.02 to 0.02 sign$(\lambda_2)\rho$) and the SAPT dispersion energy for the most stable MILs studied. EF = [Emim][$FeCl_4$], BF = [Bmim][$FeCl_4$], BFB =[Bmim][$FeBr_4$], BZ = [Bmim][$ZnCl_3$] and BS = $[Bmim]_2$[$SnCl_4$] 9

Table S1. Contributions to the interaction energy (kcal mol^{-1}) using SAPT0. Dist is the distance of the minimum interaction energy between the CM, E_{Elec} is the electrostatic contribution, E_{Exch} is the exchange contribution, E_{Ind} is the induction contribution, E_{Disp} is the dispersion contribution, E_{Tot} is the total interaction energy in SAPT0 and $E_{CCSD(T)}$ is the interaction energy with DLPNO-CCSD(T)

Molecule	Dist(Å)	E_{Elec}	E_{Exch}	E_{Ind}	E_{Disp}	E_{Tot}	$E_{CCSD(T)}$
[Bmim][$FeCl_4$](C_4–C_5)	5.341	−65.19	17.89	−5.50	−7.91	−60.71	−58.82
[Bmim][$FeCl_4$](Chain)	9.599	−35.11	5.63	−2.44	−2.40	−34.32	−33.79

Table S2. Charge of the MIL anion according to Mulliken and Natural Population Analysis (NPA) at the minimum geometry.

MIL	Q_{Mull}	Q_{NPA}
[Emim][$FeCl_4$]	−1.0620	−0.95775
[Bmim][$FeCl_4$]	−1.1317	−0.94044
[Bmim][$FeBr_4$]	−1.1196	−0.93520
[Bmim][$ZnCl_3$]	−1.0517	−0.93843
$[Bmim]_2[SnCl_4]$	−2.1194	−1.79151

Table S3. Comparison between minimum distance and energies between SAPT0 and LED-DLPNO-CCSD(T).

MIL	Method	Dist (Å)	E (kcal mol^{-1})
[Emim][$FeCl_4$]	LED	4.108	−73.441
	SAPT	4.017	−76.282
[Bmim][$FeCl_4$]	LED	4.341	−71.634
	SAPT	4.273	−73.955
[Bmim][$FeBr_4$]	LED	4.426	−71.343
	SAPT	4.342	−74.596
[Bmim][$ZnCl_3$]	LED	4.640	−70.364
	SAPT	4.574	−72.251
$[Bmim]_2[SnCl_4]$	LED	3.680	−260.076
	SAPT	3.513	−270.503

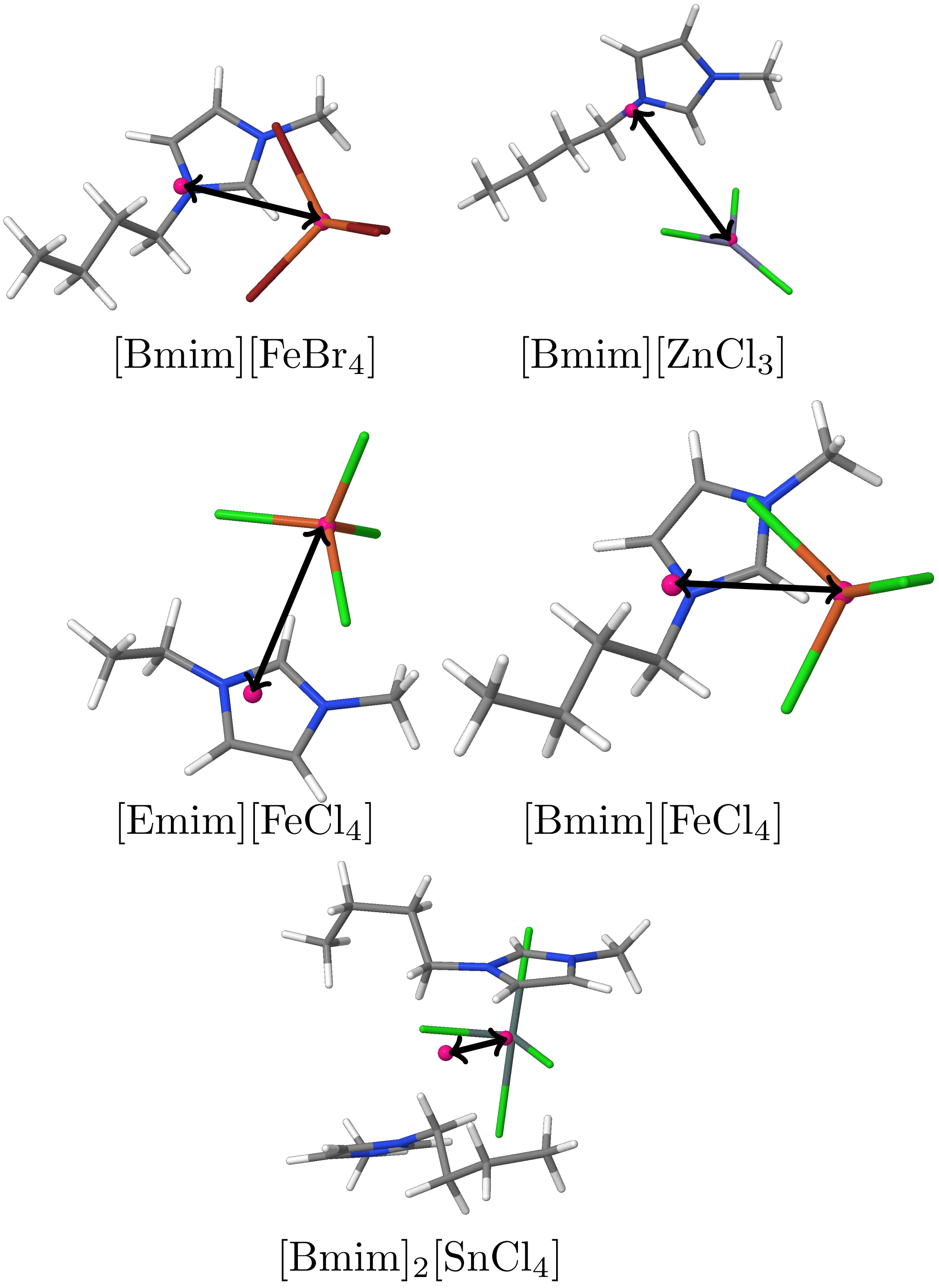

Figure S1. Distance between the centre of mass of the different monomers.

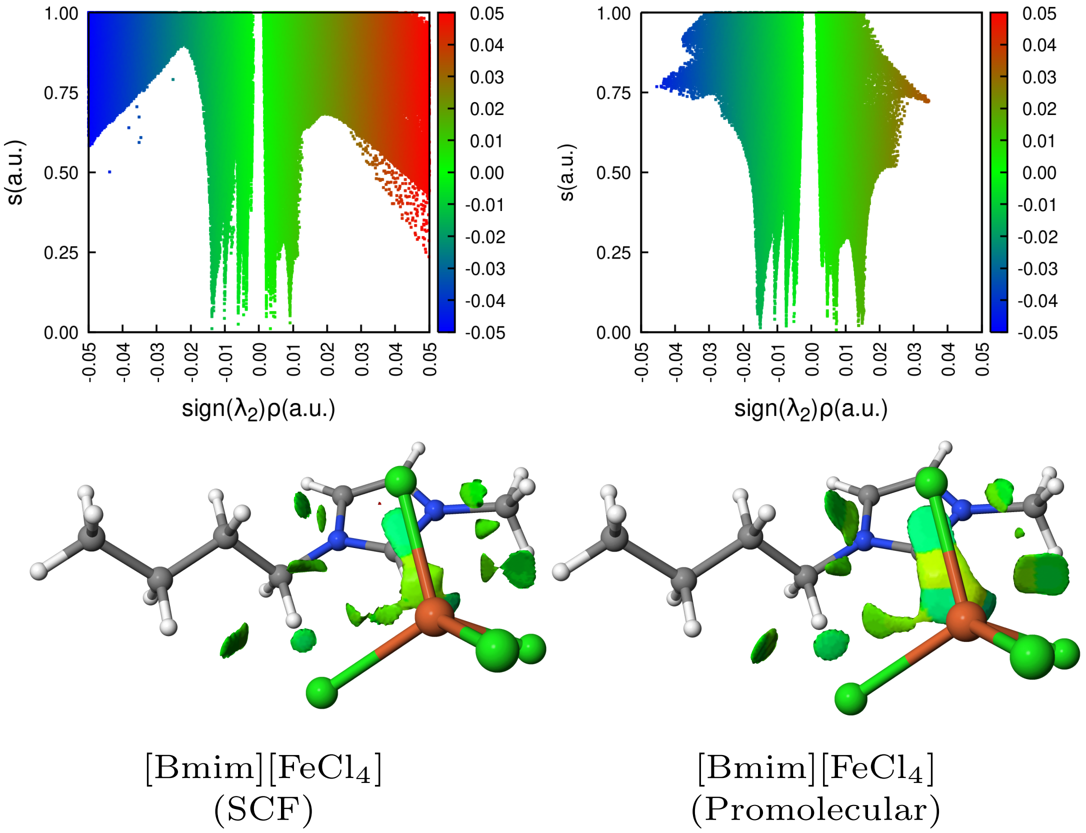

Figure S2. Comparison between SCF calculation and promolecular calculation in NCIPLOT.

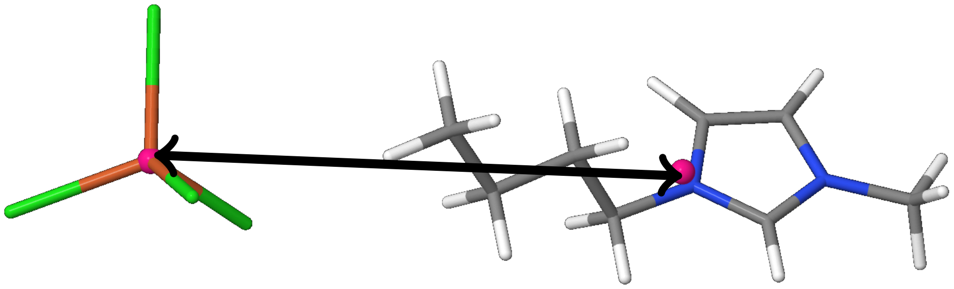

[Bmim][$FeCl_4$](Chain)

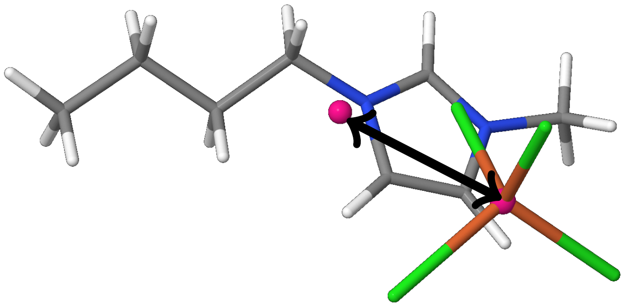

[Bmim][$FeCl_4$](C_4–C_5)

Figure S3. Distance between the centre of mass of the different additional configurations of [Bmim][$FeCl_4$]

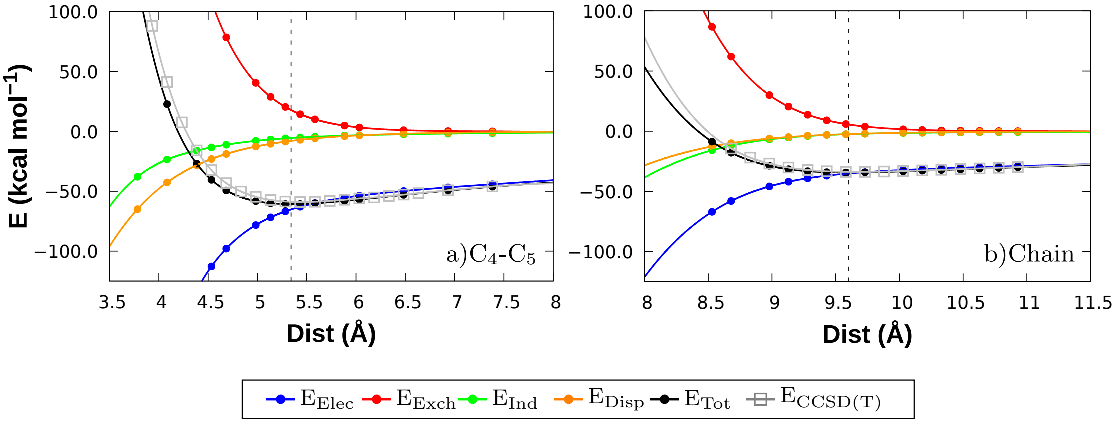

Figure S4. SAPT decomposition for an increase distance between the monomers in [Bmim][$FeCl_4$] with different dispositions.

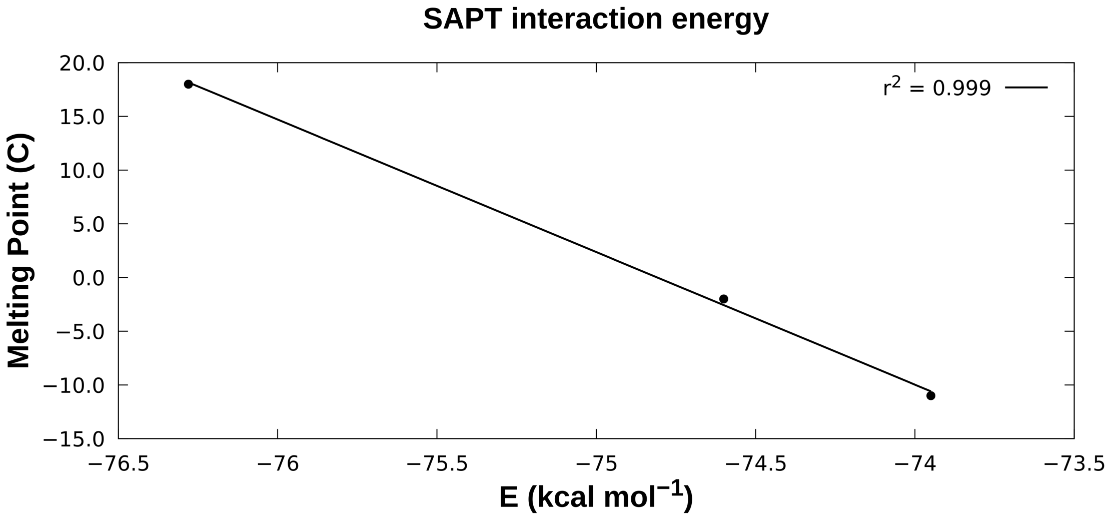

Figure S5. Relationship between SAPT energy and the experimental melting points for different MILs. Relationship between SAPT energy and the experimental melting points for different MILs.
Experimental data taken from Refs. [1,2,3].

[1] Yoshida, Y.; Otsuka, A.; Saito, G.; Natsume, S.; Nishibori, E.; Takata, M.; Sakata, M.; Takahashi, M.; Yoko, T. Conducting and Magnetic Properties of 1-Ethyl-3-Methylimidazolium (EMI) Salts Containing Paramagnetic Irons: Liquids [EMI][$M^{III}Cl_4$] (M = Fe and $Fe_{0.5}Ga_{0.5}$) and Solid $[EMI]_2[Fe^{II}Cl_4]$. Bull. Chem. Soc. Jpn. 2005, 78, 1921–1928, doi:10.1246/bcsj.78.1921.

[2] Krieger, B.M.; Lee, H.Y.; Emge, T.J.; Wishart, J.F.; Edward W. Castner, J. Ionic Liquids and Solids with Paramagnetic Anions. Phys. Chem. Chem. Phys. 2010, 12, 8919–8925, doi:10.1039/B920652N.

[3] Yoshida, Y.; Saito, G. Influence of Structural Variations in 1-Alkyl-3-Methylimidazolium Cation and Tetrahalogenoferrate(III) Anion on the Physical Properties of the Paramagnetic Ionic Liquids. J. Mater. Chem. 2006, 16, 1254–1262, doi:10.1039/b515391c.

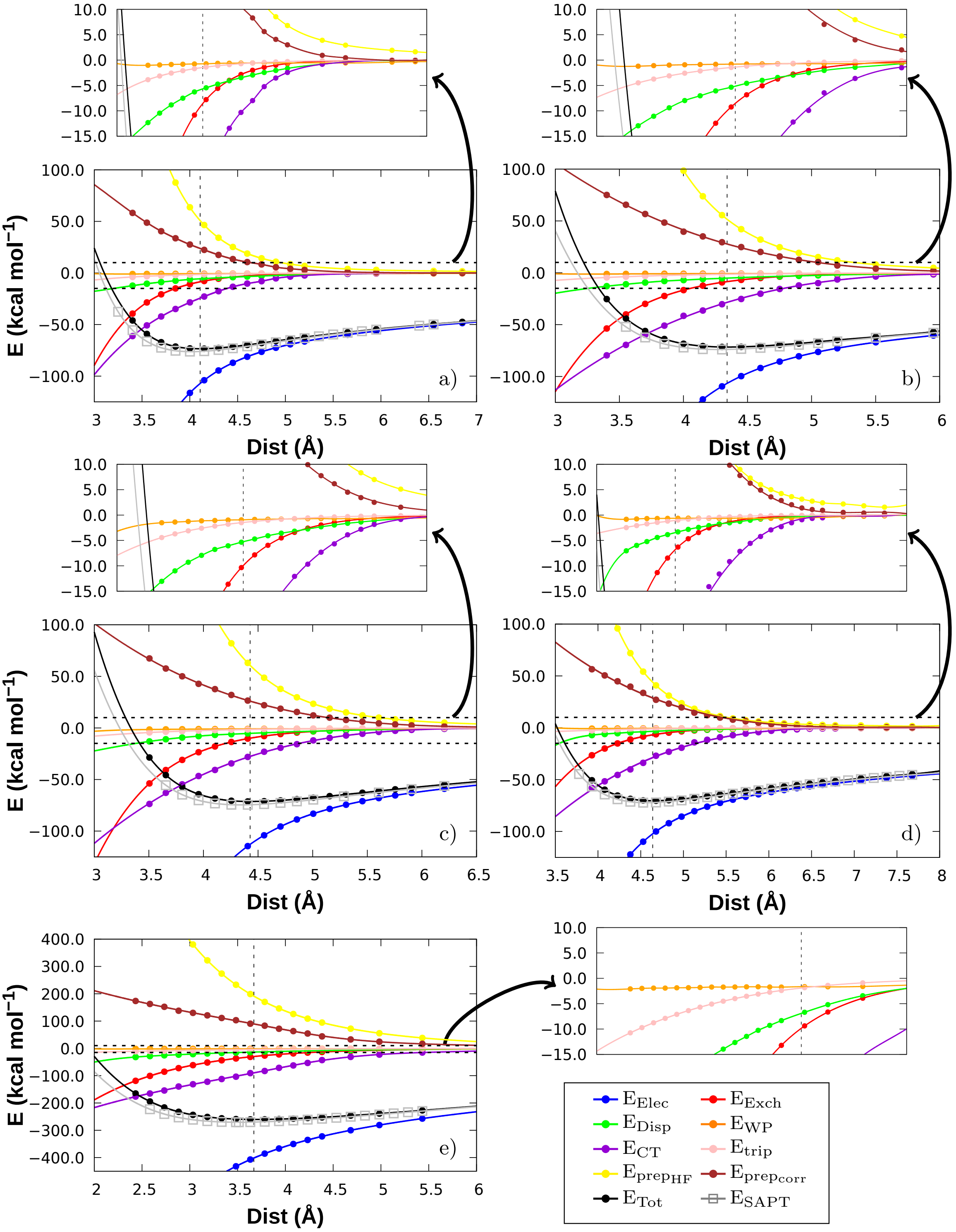

Figure S6. The LED-DLPNO-CCSD(T) decomposition for an increase distance between the monomers. The SAPT0 energy is included with the purpose to compare the results. a) [Emim][$FeCl_4$], b) [Bmim][$FeCl_4$], c) [Bmim][$FeBr_4$], d) [Bmim][$ZnCl_3$], e) $[Bmim]_2[SnCl_4]$

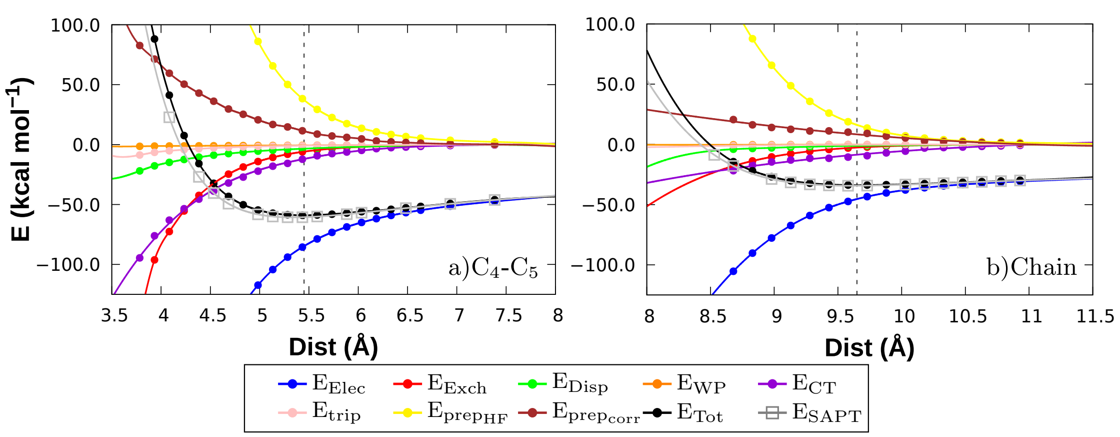

Figure S7. The LED-DLPNO-CCSD(T) decomposition for an increase distance between the monomers in [Bmim][$FeCl_4$] with different dispositions.

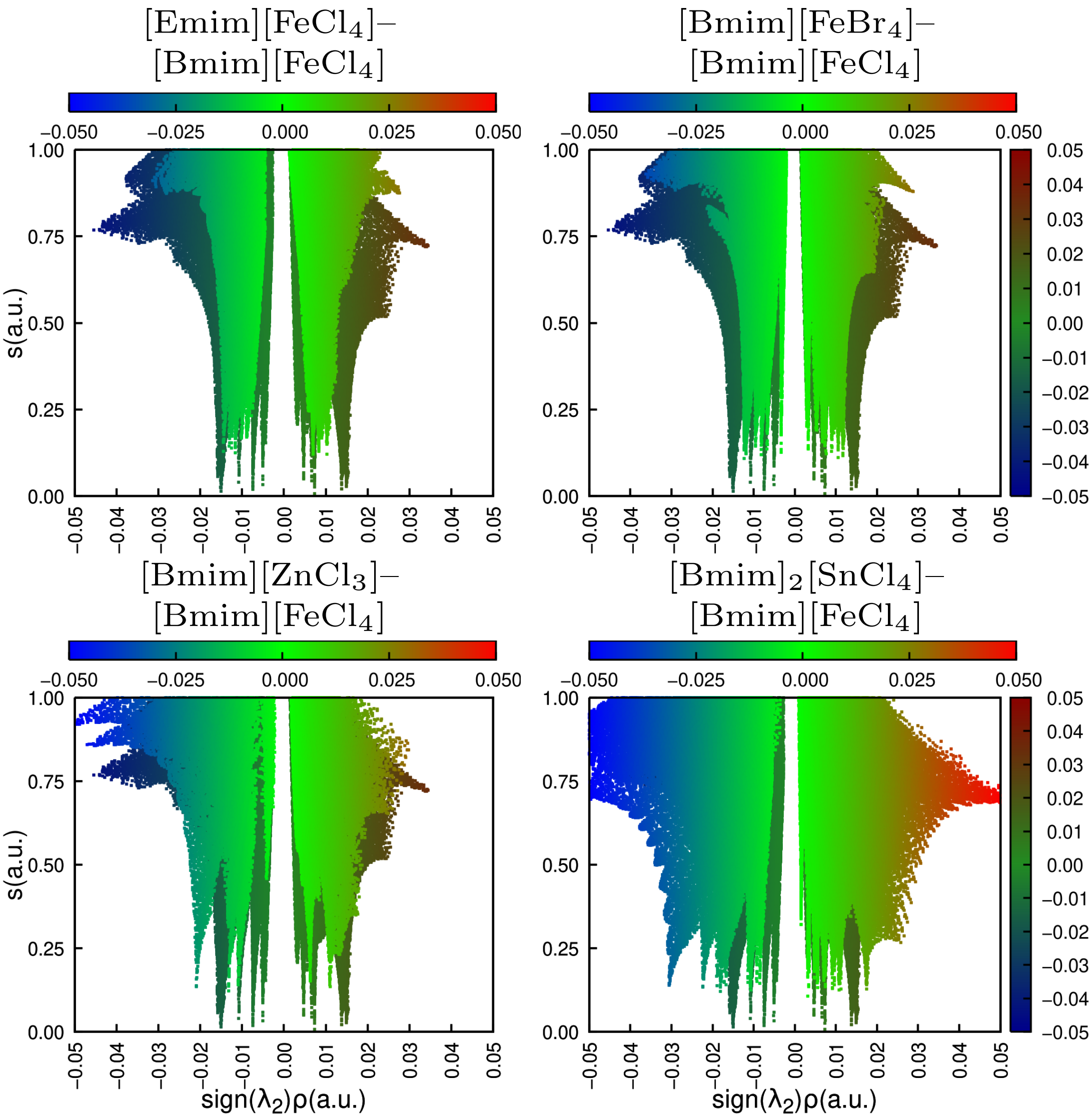

Figure S8. NCI two-dimensional plots for the MILs. The dark scale is obtained for the [Bmim][$FeCl_4$] and the other MIL is displaced 0.1 a.u. in s for clarity.

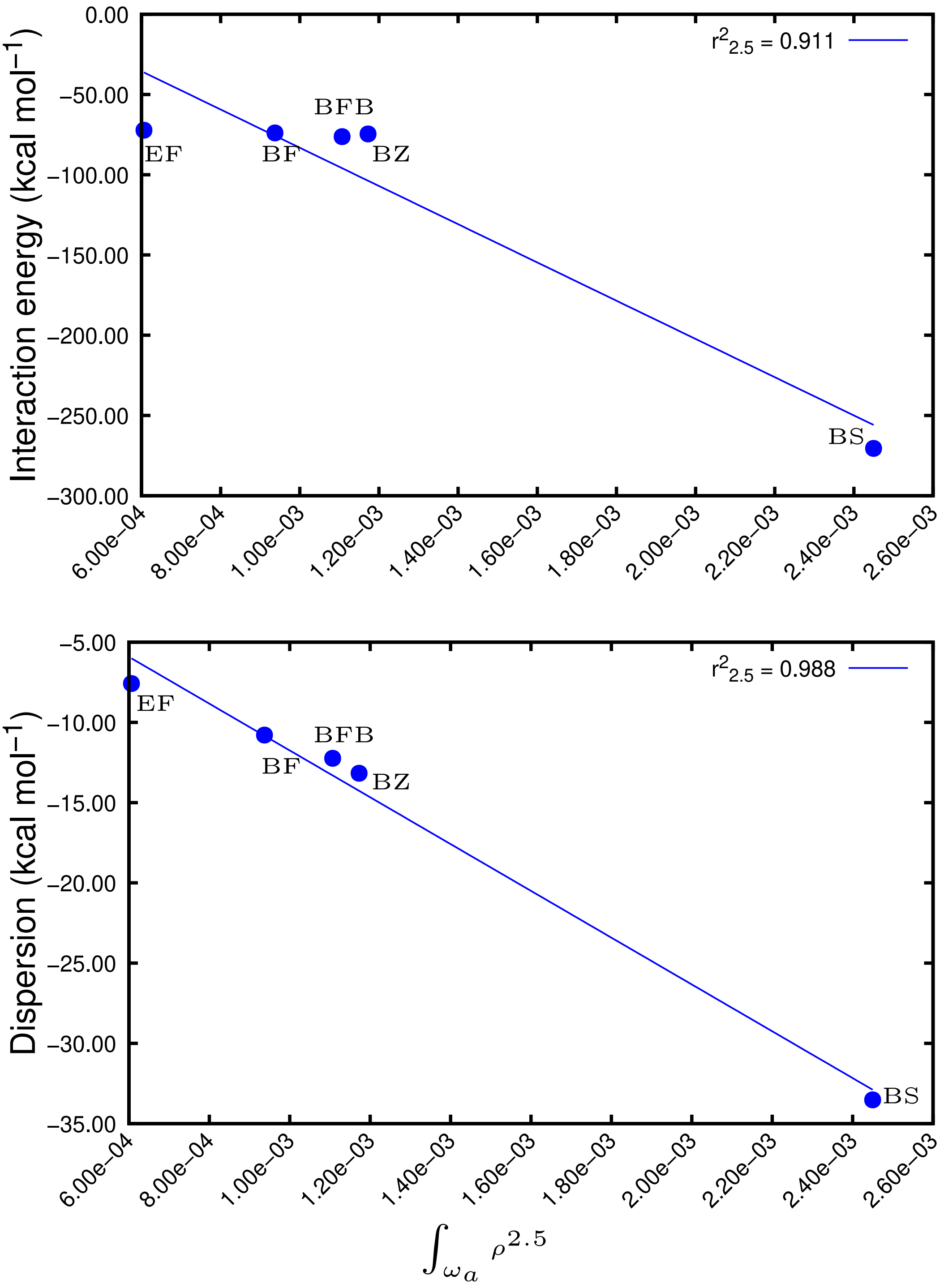

Figure S9. (top) Data fitting between the integral I2.5 and the SAPT interaction energy for the most stable MILs studied. (bottom) Data fitting between the integral I2.5 in the range values for VdW interactions (-0.02 to 0.02 sign$(\lambda_2)\rho$) and the SAPT dispersion energy for the most stable MILs studied. EF = [Emim][$FeCl_4$], BF = [Bmim][$FeCl_4$], BFB =[Bmim][$FeBr_4$], BZ = [Bmim][$ZnCl_3$] and BS = $[Bmim]_2[SnCl_4]$
